# A Molecular Dynamics Approach to Ligand-Receptor Interaction in the Aspirin-Human Serum Albumin Complex

**DOI:** 10.1155/2012/642745

**Published:** 2012-11-21

**Authors:** H. Ariel Alvarez, Andrés N. McCarthy, J. Raúl Grigera

**Affiliations:** Instituto de Física de Líquidos y Sistemas Biológicos (IFLYSIB), CONICET y Departamento de Ciencias Biológicas, Facultad de Ciencias Exactas, Universidad Nacional de La Plata, 49-789, cc 565, B1900BTE La Plata, Argentina

## Abstract

In this work, we present a study of the interaction between human serum albumin (HSA) and acetylsalicylic acid (ASA, C_9_H_8_O_4_) by molecular dynamics simulations (MD). Starting from an experimentally resolved structure of the complex, we performed the extraction of the ligand by means of the application of an external force. After stabilization of the system, we quantified the force used to remove the ASA from its specific site of binding to HSA and calculated the mechanical nonequilibrium external work done during this process. We obtain a reasonable value for the upper boundary of the Gibbs free energy difference (an equilibrium thermodynamic potential) between the complexed and noncomplexed states. To achieve this goal, we used the finite sampling estimator of the average work, calculated from the Jarzynski Equality. To evaluate the effect of the solvent, we calculated the so-called “viscous work,” that is, the work done to move the aspirin in the same trajectory through the solvent in absence of the protein, so as to assess the relevance of its contribution to the total work. The results are in good agreement with the available experimental data for the albumin affinity constant for aspirin, obtained through quenching fluorescence methods.

## 1. Introduction

Human serum albumin (HSA) is the most abundant plasma protein in the human body and plays an important role in drug transport and metabolism. Generally regarded as a nonspecific transport protein, HSA has been assigned a number of enzymatic properties [1–3]. Additionally, the enzymatic activity of HSA on different substrates and drugs has also been studied and documented. Nevertheless, the structural mechanism of this activity is yet unknown.

To assess the structural basis of binding mechanisms, we evaluated the interaction between HSA and acetylsalicylic acid (ASA, C_9_H_8_O_4_) by means of molecular dynamics simulations (MD). 

Starting from an experimentally resolved structure of the complex, we extracted the ligand by means of the application of an external force, under near quasistatic conditions, thus evaluating the work involved in breaking the interactions present in the protein-ligand complex, and hence obtaining an upper boundary for the free energy of binding of the complex.

We quantified the force used to remove the ASA from its specific site of binding to HSA and calculated the mechanical nonequilibrium external work done during this process. 

The aim of the present study is to calculate an upper boundary for the Gibbs free energy difference associated to that process, through the average work obtained using the finite sampling estimator from the Jarzynski equality. 

To evaluate the effect of the solvent (treated explicitly), we calculated the “viscous work.” This magnitude represents the work done to move the aspirin in the same trajectory through the solvent in absence of the protein. Its principal sense follows from the fact that experimental techniques to obtain the protein-ligand affinity (e.g., quenching fluorescence, microcalorimetry) do not take into account the contribution of the solvent to this out of equilibrium process (since they analyze equilibrium states).

Finally, we compared our computational results with the experimental data available for the affinity constant of the HSA-ASA complex, obtained by quenching fluorescence methods [4], showing reasonable agreement.

## 2. General Procedure

We carried out all molecular dynamics (MD) simulations using the GROMACS 4.0.1 package [5] in which the equations of motion are solved using a leap-frog integration step. We used GROMOS96 (43a1) force field [6] for the minimization process, as well as for all the MD simulation steps, and kept all protein bond lengths constrained using the LINCS algorithm [7]. Water molecules were constrained using the SETTLE algorithm [8]. For the calculation of long-range coulombic interactions, we applied the reaction field method, with a 1.4 nm cut-off radius. Likewise, we calculated Lennard-Jones interactions within a cut-off radius of 1.4 nm.

For all the simulation runs, we have used a Xeon-based, dual-processor cluster, running under GNU/Linux, and for all plots and graphics MS Windows or GNU/Linux, using the reference Visual Molecular Dynamics package, Swiss PDB Viewer, or XGrace software [9–11].

As starting configuration we used the human serum albumin-acetylsalicylic acid complex (PDB ID: 2I30-Resolution 2.9Å) that contains five myristic acid molecules (MYR, C_14_H_28_O_2_) forming part of the albumin structure [1]. Since the original structure was incomplete, we reconstructed both the N-terminus and the C-terminus. Thus, ASP1, ALA2, and LEU585 had to be added *ad hoc* to the experimental structure using Swiss PDB Viewer [12], using standard *ϕ* − Ψ angles for the reconstructed peptidic planes. Moreover, the experimental structure corresponds to the complex in the post reaction state; that is, the protein was acetylated in residue LYS199 and the aspirin hydrolyzed. Thus, we removed the acetyl group from the protein and reconstructed the modified aspirin.

Having focused on the interaction (and dissociation) between HSA and its most internal (and more tightly attached) aspirin ligand, such process will be expected to occur in absence of the less tightly bonded (and external) aspirin ligand. Consequently, the external aspirin was removed from the initial configuration.

The starting system consisted of a truncated dodecahedral simulation box of dimension parameter *d* = 12.9702 nm and a total volume of 1540.65 nm^3^, containing one HSA molecule, one ASA molecule, five myristic acid molecules, and 47.803 water molecules. We used the SPC/E [13] water model. We aligned the principal axes of the protein and the box.

We constructed the topologies for aspirin and myristic acid and added them to GROMOS96 43a1 force field residue data base, using all the corresponding atom types, charges, bonds, angles, proper, and improper dihedral angles from that database. The values used for all atom charges in both aspirin and myristic acid topologies are summarized in Tables 1a and 1b. A detailed schematic representation for both molecules is additionally provided as supplementary material (see Supplementary Material available online at doi:10.1155/2012/642745). As previously validated in recent literature [14], myristic acid was modeled in the unprotonated state and described using parameters derived from the lipid force field [15]. The carboxylic acid group was based on the parameters of glutamic acid, which were available from the corresponding GROMOS96 43a1 force field.

We generated the topology of the total system, with standard protonation states for all amino acids (pH 7). 

In every step, we removed the motion of the center of mass of the system.

The production system was weakly coupled to a thermal and hydrostatic bath, in order to work in the isothermal-isobaric ensemble [16] at *T* = 300 K and *P* = 1 bar.

## 3. Equilibration

We minimized the energy of the system using firstly the steepest descent method, converging to machine precision. Secondly, we applied the conjugated gradient method, converging in less than 20 cycles.

The equilibration of the complete system proved to be nontrivial, because we had to complete the original structure and chemically modify it, and we had to perform a nonstandard series of computational steps to release the exogenous tensions introduced due to the structural additions and modifications mentioned previously. Namely, we performed an MD run *in vacuo* (in absence of solvent), using a 0.0001 fs time step and for a total time of 2 ps lightly coupled to a 100 K temperature bath [16], after energy minimization in order to let the incorporated residues explore more stable configurations. During this run, we applied position restrains on all of the backbone atoms (except for those incorporated *ad hoc*). In the same conditions, we subsequently performed a 10 ps run, using a 0.5 fs time step. Next and for another 10 ps (using the same time step), we incorporated the LINCS algorithm to add restrictions in bond lengths and angles for all molecules belonging to the complex.

Once the previous procedure was completed, we gradually increased the time step up to 2 fs, likewise increasing temperature up to the final value of 300 K.

At this point, we solvated the system adding 47.803 SPC/E explicit water molecules. In these conditions, we allowed the solvated system to relax (keeping all solute heavy atoms restrained to their corresponding crystallographic positions) during 200 ps, at 300 K and 1bar. At the same temperature and pressure, we subsequently decreased the position restraints force constants during a 200 ps MD run. Finally, we released all position restraints and allowed the system to relax freely for a total simulation time of 20 ns, at 300 K and 1bar, after which we considered the system to be in conformational equilibrium.

The stability of MYR molecules in their binding sites throughout the total 20 ns of the equilibration run indicates that the conformational equilibrium was correctly achieved. Together with this, we observed a relative stability of the HSA backbone (evaluated by the convergence of HSA alpha-carbon root mean square displacement (RMSD), (see Supplementary Material available online at doi:10.1155/2012/642745)). These facts are in notable agreement with the widely known role of fatty acids in the stabilization of a HSA structure [17]. 

## 4. AFM Pulling Simulation

Once the system was effectively equilibrated, we performed 30 MD quasistatic runs, applying an external force on the center of mass of the aspirin, in order to extract this molecule from its binding site. We coupled all 30 production simulation runs to a weak temperature and pressure bath of 300 K and 1 bar, in order to work in the isothermal-isobaric ensemble. We randomly generated the initial velocities, using a unique seed for every production run. The total simulation time of every production run was 500 ps, with a time step of 2 fs. The constant of the harmonical potential applied at the center of mass of the aspirin was of *k* = 1, 000 kJ/(mol·nm^2^), and the pulling rate (i.e., the velocity used to move the free end of the “*spring*” in the pulling direction) was *v* = 0.01 nm/ps.

The pulling direction was established as the line connecting the center of mass of the ligand and the center of the triangle determined by the centers of mass of the R groups of residues GLU 292, HIS 440, and CYS 448 (i.e., the binding pocket threshold).

**Figure 1:**
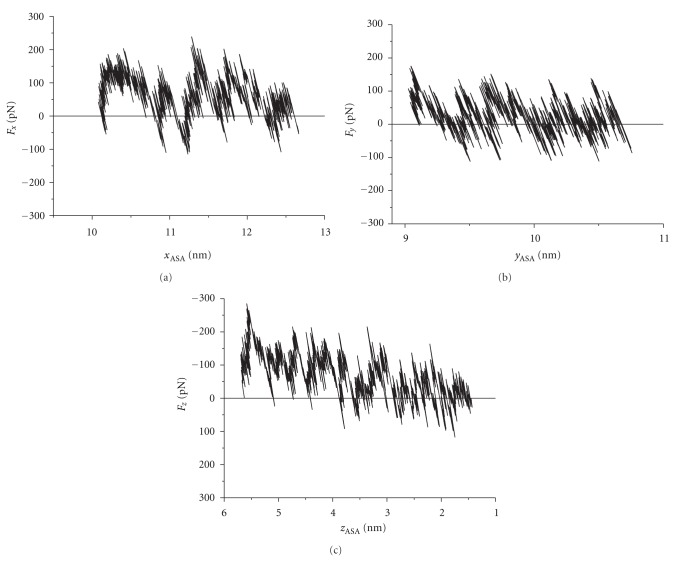
Example of the components *x*, *y*, and *z* of the applied force, as a function of the respective position component of the acetylsalicylic acid molecule center of mass (Figures (a), (b), and (c), resp.). The displacement in the *z* axis was in the negative direction, while for *x* and *y* axes the displacement was in the positive direction.

To calculate the work done during this process, we plotted each component of the force (*F*_*x*_, *F*_*y*_, *F*_*z*_) as a function of the respective position component of the center of mass of the acetylsalicylic acid molecule (*x*_ASA_, *y*_ASA_, *z*_ASA_). This is shown in Figure 1. These graphics show resemblance to the experimental data obtained from Atomic Force Microscopy [18].

## 5. The Jarzynski Relationship

The process of removing a ligand from the site of binding to its receptor can be done by applying an external force to the ligand, hence performing a corresponding external mechanical work. If this work is done quasistatically, the value obtained for the applied external work is equal to the free energy variation associated to that process.

When the work done is not quasistatic, the Jarzynski equality [19, 20] gives a relationship between the variation in free energy values that describes the state changes in the system under study, and the correctly averaged ensemble of the measurements of the external work done to change that state in a finite time (out of equilibrium). In the case of a system in contact to an external thermal bath (in the Canonical ensemble), the correct description of the state changes of the system is through Helmholtz free energy, whereas for a system in the isothermal-isobaric ensemble like the one we are studying, the suitable descriptor of the equilibrium states is Gibbs free energy. 

If the actual distribution is approximated by performing a finite sample of work performed through an applied external force, a superior value to the free energy variation can be obtained from the average work *W* ^*a*^ of the finite sampling, taken as usual:

(1) $$\begin{array}{c} W^{a}\equiv \frac{1}{N_{s}}\sum_{i=1}^{N_{s}}W_{i}, \end{array}$$

where *N*_*s*_ is the number of measurements and *W*_*i*_ the external work done in the *i*th process. We can think of *W*_*i*_ as a random sampling from the distribution *ρ*(*W*) that satisfies the so-called Jarzynski equality at any time *t*_*s*_:

(2) $$\begin{array}{c} \langle e^{-W/k_{B}T}\rangle \equiv \int W\rho \left(W,t_{s}\right)e^{-W/k_{B}T}dW=e^{-\Delta G/k_{B}T}, \end{array}$$

where *T* is the temperature, *k*_*B*_ the Boltzmann constant, *t*_*s*_ is the time spent to do the described process, and Δ*G* is the Gibbs free energy variation.

In this context, the expectation value of *W* ^*a*^,  〈〈*W* ^*a*^〉〉, will give an upper boundary to the Gibbs free energy variation:

(3) $$\begin{array}{c} \langle \langle W^{a}\rangle \rangle =\langle W\rangle \equiv \int W\rho \left(W,t_{s}\right)dW\geq \Delta G. \end{array}$$

The Jarzynski equality suggests that *W* ^*a*^ is not the correct quantity to estimate Δ*G*. A better upper boundary to this magnitude is given by the so-called finite sampling estimator of the average from the Jarzynski equality [21]:

(4) $$\begin{array}{c} W^{x}\equiv -k_{B}T\cdot \ln\left[\frac{1}{N_{s}}\sum_{i=1}^{N_{s}}e^{-W_{i}/k_{B}T}\right]. \end{array}$$

For *N*_*s*_ = 1, *W* ^*x*^ and *W* ^*a*^ will be equal and the expectation value for both will be 〈*W*〉, and for *N*_*s*_ → *∞*, *W* ^*x*^ will tend to Δ*G*, and *W* ^*a*^ will tend to 〈*W*〉.

For intermediate values of *N*_*s*_,

(5) $$\begin{array}{c} \Delta G\leq \langle \langle W^{x}\rangle \rangle \leq \langle \langle W^{a}\rangle \rangle , \end{array}$$

is satisfied.

## 6. The Viscous Work

The data obtained through nonequilibrium molecular dynamics pulling of a ligand may reasonably contain a nondesired contribution due to the work that is necessary to move a molecule through the solvent under such conditions, when compared to experimental data obtained in conditions of equilibrium. Hence, we have undertaken the evaluation of the relevance of the so-called “viscous work,” in comparison with the total work.

With this purpose, we prepared a system of the same shape and dimensions, consisting only of one acetylsalicylic acid molecule in the original position, solely surrounded by water molecules.

Firstly, we minimized the energy of this system as described in the previous section and, subsequently, performed short 200 ps stabilization. Once temperature and pressure reached equilibrium values of 300 K and 1 bar, we coupled the system to a weak thermostatic bath and fixed the box dimensions. After stabilization, we proceeded as with the system in presence of HSA, performing 30 MD runs with the same general setup.

## 7. Results and Discussion

**Figure 2:**
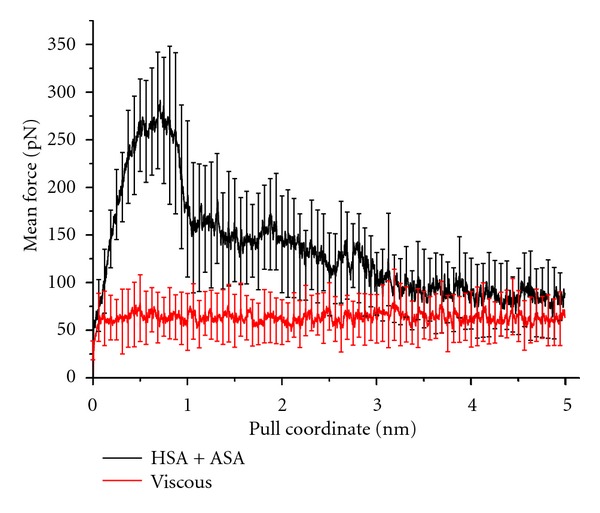
Dependence of the value of the magnitude of the mean force applied on the center of mass of ASA as a function of the magnitude of the displacement of the spring (pull coordinate), both for the system in presence (black lines) and absence (red lines) of the HSA complex. These values represent the mean force at each step, averaged over the thirty MD runs for both systems. The error bars show the standard deviation of these mean values (only 80 values are here presented in order to enhance graphical clearness).

The data presented in Figure 2 corresponds to the mean value of the magnitude of the force applied on the center of mass of the acetylsalicylic acid molecule, as a function of the magnitude of the displacement of the free end of the ideal spring, both for the system in presence (black lines) and absence (red lines) of the HSA complex. Error bars show the standard deviation of those values. In order to obtain greater graphical clearness, only 80 of these bars are shown. In this Figure, a marked difference between curves may be observed up to 2 nm. After this point the error bars cross each other, finally reaching statistical convergence. The duplication of this distance (the point of error bar crossing, that is, 4 nm) was considered as a reasonable criterion for convergence and was thus chosen as the limit value for production data. After this point, the magnitude of the applied force becomes comparable for both systems, which allows us to consider the acetylsalicylic acid molecule to be out of the binding site.

**Figure 3:**
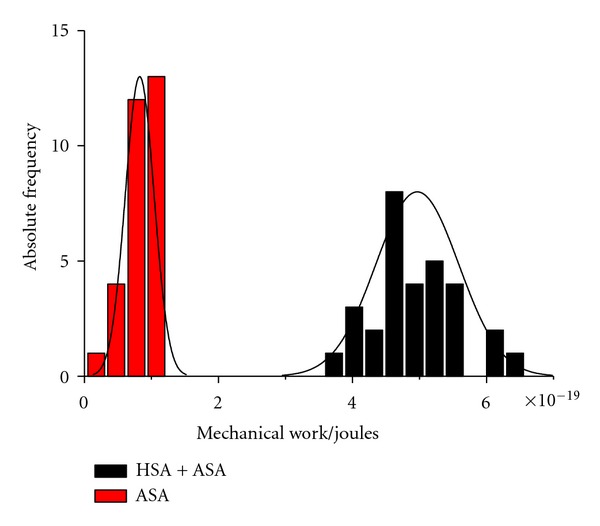
Histogram representation of the mechanical work, both for the system in presence (black bars) and absence (red bars) of the HSA complex. The continuous lines are the Gaussian distributions for the corresponding values of standard deviation and mean value for each histogram population.

In Figure 3, we present a histogram representation of the distribution of the performed mechanical work, both for the system in presence (red bars) and absence (black bars) of the HSA complex. From this study, we may interestingly observe that the studied samples of both systems are large enough to clearly distinguish between populations. 

The obtained values for *W* ^*a*^ and *W* ^*x*^, using (1) and (4), respectively, are

(6) Wa=(5.0±0.6)×10−19 J (usual  average),Wx=(4.0±0.6)×10−19 J (Jarzynski's  average).

From these results we can observe that *W* ^*x*^ establishes a better highest value for Δ*G* than *W* ^*a*^, in agreement with (5). 

Likewise, the average value of the viscous work obtained using the usual average is

(7) $$\begin{array}{c} {W_{}^{a}}_{\text{viscous}}=8\pm 2\times 10^{-20}\;\text{J}. \end{array}$$

And for the finite sampling estimator of the average value from Jarzynski equality,

(8) $$\begin{array}{c} {W_{}^{x}}_{\text{viscous}}=4\pm 2\times 10^{-20}\;\text{J}. \end{array}$$

For the present case, the obtained value of the viscous work falls within the order of magnitude of the standard deviation of the calculated binding work. However, this may not be the general situation. Additionally, as such computational methods are refined and sampling capacities increased, this “viscous work” may reasonably become a more important source of discrepancy with experimental observation, therefore rendering its evaluation mandatory.

Taking these into account, the obtained value for *W* ^*x*^ would be

(9) $$\begin{array}{c} {W_{}^{x}}_{\text{corrected}}=\left(3.6\pm 0.6\right)\times 10^{-19}\;J. \end{array}$$

Regarding the ligand trajectory in this process, we can distinguish two regions: an interior region that corresponds to the movement within the binding pocket, where the pulling direction is univocally determined by the shortest path between the original position of the ligand and the binding pocket threshold and a second exterior one.

In the first region, where the unbinding path is conformationally determined, we can assume that the contribution to the total average work is close to the optimal one, whereas in the exterior region it is expected that better path optimization may be achieved.

The separation between both regions was established as the point in which the center of mass of the ligand crosses the plane formed by residues GLU 292, HIS 440, and CYS 448 (i.e., the binding pocket threshold).

The first region represents 29.4% of the total trajectory, with a corresponding pulling coordinate of 1.47 nm, and its contribution to the corrected work is of (1.9 ± 0.6) × 10^−19^ J. 

The experimental affinity constant of the complex HSA-aspirin, obtained by Bojko and coworkers from quenching fluorescence measurements [4], is *K*_*a*_ = 18.79 × 10^3^ M^−1^. From this value, we obtain the experimental Δ*G* as

(10) $$\begin{array}{c} \Delta G=k_{B}T\ln K_{a}=4.07\times 10^{-20}\;\text{J}. \end{array}$$

The upper boundary obtained using the finite sampling estimator of the average work gives a better value than that obtained from the usual average work. Albeit more recent comments on Jarzynski's work [22], his response is clear and categorical [23], and this result comes in line with what he classically proposes.

## 8. Conclusion

In the present study, we have succeeded in reconstructing, stabilizing, and equilibrating a protein ligand complex which proceeded from incomplete data provided from experiment. This achieved equilibrium state enabled us to study the HSA-ASA-MYR complex through nonequilibrium molecular dynamics, in near quasistatic conditions. The fact that complex biological structures, that are not totally resolved through experiment, may be eligible targets for molecular dynamic studies reinforces the importance and potentiality of this technique.

We have likewise obtained an average upper boundary for the Gibbs free energy of binding of the HSA-ASA complex that is less than one order of magnitude above the experimental value. 

As regards the assessment of the relevance of the viscous work, the value obtained is low compared to the upper boundary for the Gibbs free energy difference of the HSA-ASA complex. Nevertheless, its difference with the experimental free energy of binding is not negligible and should therefore be generally taken into account in such studies. Moreover, the relevance of this effect, and the need for its correct evaluation may reasonably increase as computational methods are refined and sampling capacities increased.

Finally, the promising level of agreement of the data presented in this study with that available experimentally calls for further development of such studies as well as encourages the analysis of other similar systems.

## Supplementary Material

Fifty nanoseconds long HSA alpha-carbon root mean square displacement plot, and an schematic 2D representation of both Acetylsalicylic Acid and Myristic Acid, showing the topologies' construction.

## Figures and Tables

**Table 1a:** (a)

Atom number	Atom Type	Name	Charge group	Partial Charge	Mass
1	OM	OL	1	−0.635	15.9994
2	C	CK	1	0.27	12.011
3	OM	OM	1	−0.635	15.9994
4	C	CJ	2	0	12.011
5	CR1	CI	2	0	13.019
6	CR1	CH	2	0	13.019
7	CR1	CG	3	0	13.019
8	CR1	CF	3	0	13.019
9	C	CE	3	0	12.011
10	OA	OD	4	0	15.9994
11	C	CB	4	0.38	12.011
12	O	OC	4	−0.38	15.9994
13	CH3	CA	5	0	15.035

**Table 1b:** (b)

Atom number	Atom Type	Name	Charge group	Partial Charge	Mass
1	OM	OB	1	−0.635	15.9994
2	C	CA	1	0.27	12.011
3	OM	OC	1	−0.635	15.9994
4	CH2	CD	2	0	14.027
5	CH2	CE	2	0	14.027
6	CH2	CF	2	0	14.027
7	CH2	CG	2	0	14.027
8	CH2	CH	2	0	14.027
9	CH2	CI	2	0	14.027
10	CH2	CJ	2	0	14.027
11	CH2	CK	2	0	14.027
12	CH2	CL	2	0	14.027
13	CH2	CM	2	0	14.027
14	CH2	CN	2	0	14.027
15	CH2	CO	2	0	14.027
16	CH3	CP	2	0	15.035
