# Supplementary material for: A Molecular Dynamics Approach to Ligand-Receptor Interaction in the Aspirin-Human Serum Albumin Complex
Source: J Biophys. 2012 Nov 21;2012:642745. doi: 10.1155/2012/642745 (PMC3512314; doi:10.1155/2012/642745)
Supplement: Supplementary file 1 — Fifty nanoseconds long HSA alpha-carbon root mean square displacement plot, and an schematic 2D representation of both Acetylsalicylic Acid and Myristic Acid, showing the topologies' construction. [file 642745.f1.pdf]

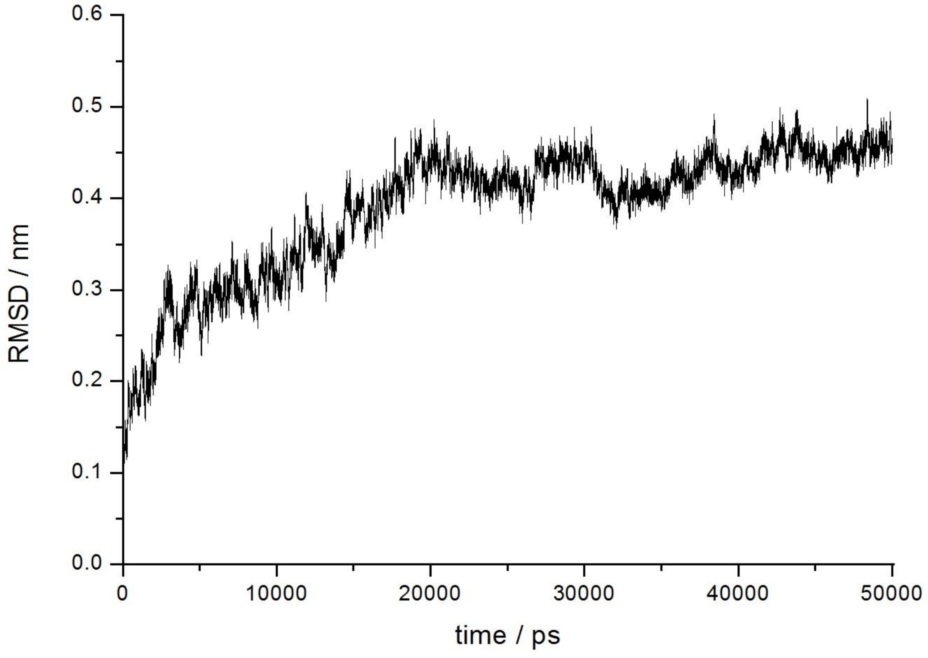

Supplementary Material 1 – 50ns HSA alpha-carbon root mean square displacement (RMSD)

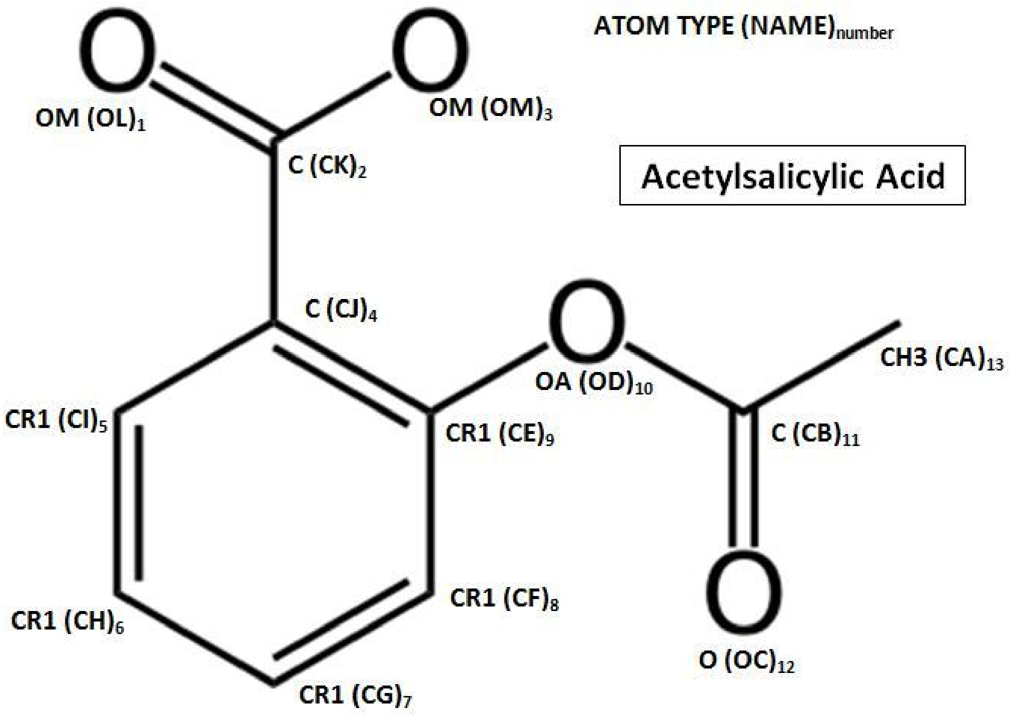

Supplementary Material 2 – Schematic 2D representation of Acetylsalicylic Acid

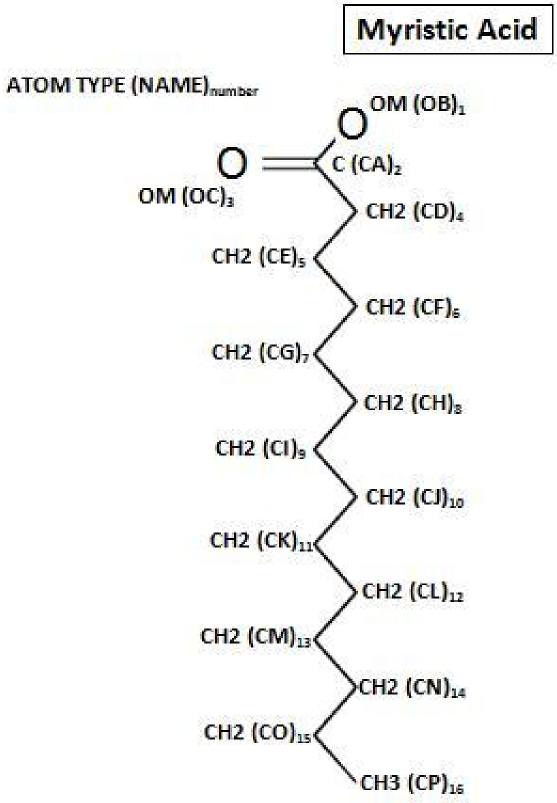

Supplementary Material 3 – Schematic 2D representation of Myristic Acid
